# Reconstructing the History of Yeast Genomes

**DOI:** 10.1371/journal.pgen.1000483

**Published:** 2009-05-15

**Authors:** David Sankoff

**Affiliations:** University of Ottawa, Ottawa, Ontario, Canada; University of Michigan, United States of America

Some 12 years ago, Wolfe and colleagues demonstrated that *Saccharomyces cerevisiae* is the descendant of an ancient whole-genome duplication event [1],[2], much to the consternation of many of those who had recently completed the sequencing of this yeast [3], the first eukaryotic nuclear genome to be sequenced. Despite persistent rejectionist argument [4], this breakthrough discovery has been amply confirmed [5],[6] and has been the starting point for scores of papers on yeast evolution and phylogeny, culminating in the Yeast Gene Order Browser [7] and the paper by Gordon et al. in this issue of *PLoS Genetics* [8].

Conceptually, the phylogenetic study of gene content, including gene gains and losses, does not depend on gene order considerations. Indeed, a preliminary step in the method of Gordon et al. is the inference of the gene content at the ancestral nodes of the assumed phylogenetic tree of 11 yeast species. Since spatial proximity of functionally interacting genes on chromosomes is relatively less important than in prokaryotes, the evolution of function would not seem to require knowledge of gene order changes. However, as is abundantly illustrated in the Research Article [8], syntenic information is crucially useful in many ways, such as: (1) confining the evolutionarily most volatile parts of the genome to subtelomeric regions, allowing the rest to be analyzed with great confidence; (2) identifying the location of the original member of dispersed gene families; (3) detecting the orthologies of fast-evolving genes; (4) identifying true gene gains (orphan genes and families); and (5) showing which genes arose from transposable elements and demonstrating the domesticated status of certain of these genes. These types of results are primarily important for the accurate reconstruction of functional evolution. At the same time, of course, this work yields much information about structural evolution, such as the enrichment of breakpoints of chromosomal rearrangement for tRNA genes and origins of replication, a parallel enrichment of gene gain sites, and a relatively low breakpoint re-use rate.

Although rearrangement-based phylogenies for mammals, where coding sequence represents but a small proportion of the genome, have been constructed based on banding patterns [9], genomic sequence [10], and everything in between, for high-resolution analyses, complete sequences, including the relatively rapidly evolving intergenic regions, should be used. For gene-dense eukaryotic genomes such as those of *Drosophila* [11] or *Saccharomyces* [8], however, gene order data represent the best compromise between maximum coverage of the genome and maximum confidence in the orthology identifications.

Rearrangement phylogeny is a very active field in computational biology. Despite the availability of many accurate and rapid algorithms, Gordon et al. have wisely and courageously chosen a manual approach to reconstruct the ancestral genomes, comparing corresponding regions in the data genomes in overlapping 25-gene windows, and resorting to trial and error inference of events, breakpoints, and conserved regions to arrive at a locally parsimonious solution; courageous because of the great amount of tedious work involved, and wise because of current deficiencies of automated approaches. First, there are generally large numbers of rather different optimal ancestors under the same objective criterion. Increasing the number of related species in the dataset without increasing phylogenetic time-depth can attenuate this, but only to a limited extent. Second, automated methods are unable to circumscribe or take into account, on the fly, genomic regions where mapping or orthology decisions may be equivocal, without the constant intervention of an expert annotator. In the Gordon et al. study, the delimitation of the subtelomeric regions to be excluded from the analysis required highly informed scientific judgment to make the trade-off between increased coverage and increased uncertainty. Third, computer programs suffer from both simplistic objective functions and overly constrained models of gene order change, both of which can lead to misleading results. For example, Gordon et al. identified a class of “telomeric translocations,” a recurrent type of rearrangement operation that is not part of the standard repertoire of rearrangement operations—namely inversions, reciprocal translocation, chromosome fission, chromosome fusion, and, in some models, unrestricted transposition or interchange of chromosomal segments. Existing algorithms would account for each telomeric translocation using a combination of standard rearrangements at increased cost, and so realistic pathways including this operation would be downgraded, because they are too expensive.

Nevertheless, there is reason to be optimistic that with the lessons learned from the manual reconstruction exercise, automated methods will eventually approach the accuracy of expert reconstruction. “Guided genome halving” currently slashes the ambiguity involved in reconstructing ancestral whole-genome duplication events by situating this ancestor in phylogenetic context, based on natural definitions for rearrangement distances among both diploid and polyploidy genomes [12]. Algorithmicists and empiricists converge on the same analytical devices: consider Figure 4 in Gordon et al. [8] and the natural adjacency graphs they cite in Warren's and Mixtacki's work. The mutual leveraging of orthology identification and syntenic block construction is a common theme in both empirical and algorithmic work.

Gordon et al. report that breakpoint re-use is 1.22 per breakpoint site, which is quite low compared to values between 1.6 and 1.9 published for mammalian genomes. Instead of relying on the following formula: reuse = twice the number of rearrangements/number of breakpoints [13], they actually looked at each site to see whether it was re-used in the evolutionary trajectory between the ancestor and *S. cerevisiae*. There are many difficulties in interpreting breakpoint re-use calculations. First, many of the rearrangements have a telomere as one of the breakpoints, and it is not at all clear whether these should be counted as full breakpoints, as not breakpoints at all, or something in between [14]. If they are not full breakpoints, this will artificially inflate the re-use rates. Second, if re-use rate is meant to be a property of a phylogenetic domain—such as hemiascomycetes yeast, mammals, or *Drosophila*—then the re-use value should be fairly constant within any subdomain and should not depend on the time-depth of the subdomain. But in reality, re-use rates increase with increasing time depth [15], which is not at all consistent with an invariant property of a phylogenetic domain. Third, if the rearrangement operations that actually generated the data are not the standard inversions, translocations, fusions, and fissions, this can affect the re-use calculation. Fourth, if an endpoint of two inversions or translocations falls in a large intergenic region between two genes, it becomes less clear whether this should be counted as the same breakpoint. This decision directly affects the calculation of breakpoint re-use. Fifth, if there are substantial genomic regions that are excluded from the analysis, such as the subtelomeric regions in the Gordon et al. paper, this can be a serious source of error in calculating rearrangement distance, breakpoints, and re-use. Finally, there is reason to believe that breakpoint re-use is simply a measure of the deterioration of the evolutionary signal contained in gene order [16].

Out of all the species studied in this paper, the detailed accounting of functional consequences at the gene gain and loss has focused on *S. cerevisiae*. This is largely due to greater amount of biological knowledge about this species. But many of the structural analyses could be repeated for all of the data species, allowing a solid assessment of the quantitative parallels and differences in evolutionary patterns across this phylogenetic domain.
